# Effect of the Characters of Chitosans Used and Regeneration Conditions on the Yield and Physicochemical Characteristics of Regenerated Products

**DOI:** 10.3390/ijms16048621

**Published:** 2015-04-17

**Authors:** Chu Hsi Hsu, Szu Kai Chen, Wei Yu Chen, Min Lang Tsai, Rong Huei Chen

**Affiliations:** 1 Department of Food Science, National Taiwan Ocean University, 2 Pei-Ning Road, Keelung 20224, Taiwan; E-Mails: chuhsi@mail.ypu.edu.tw (C.H.H.); skyskchen@gmail.com (S.K.C.); walkergky@yahoo.com.tw (W.Y.C.); 2 Department of Food and Beverage Management, Yuanpei University of Medical Technology, 306, Yuanpei Street, Hsinchu 30015, Taiwan

**Keywords:** chitosan, regeneration, molecular weight, polydispersity index, degree of deacetylation, crystallinity, ash content

## Abstract

The objective of this study was to explore the effect of the character of chitosans used, and the regeneration conditions employed on, the yield and physicochemical characteristics of regenerated products. Different concentrations of acetic acid were used to dissolve chitosans of 61.7% and 94.9% degree of deacetylation (DD), and weight-average molecular weight (*M*w) of 176 and 97 kDa, respectively; they were then precipitated with an 8 N NaOH solution, followed by washing and neutral and freeze drying to get the regenerated products. Yields of regenerated products and their physicochemical properties, such as ash content, bulk density, *M*w, polydispersity index (PDI), DD, and crystallinity were measured. A higher concentration of acetic acid used resulted in a higher yield. The purity of the regenerated product increased significantly, whereas the bulk density and crystallinity decreased significantly after regeneration. The regeneration process showed its merits of narrowing down the PDI of regenerated products. The DD and structure of chitosan was changed insignificantly after the regeneration process.

## 1. Introduction

Chitosan is a high molecular weight (*M*r) polysaccharide and is composed of *N*-acetyl-d-glucosamine and d-glucosamine, linked by β (1→4) glucoside. It is widely distributed and readily available, with the special features of being a cationic polyelectrolyte in an acid solution, non-toxic, and biodegradable. Chitosan is considered to be both a versatile and environmentally friendly raw material, and can be applied in food processing, agriculture, biomedicine, biochemistry, cosmetics, textiles, and wastewater treatment [1,2,3,4]. This is due to their versatile forms of fibers, hydrogels, membranes, microspheres, microcapsules, nanoparticles, liquid crystalline, *etc.* [5,6,7,8].

Medical and health care products, such as wound dressings, gauzes, and *in vivo* absorbable sutures, have the highest market value among the products sold in the above-mentioned areas [9]. Hirano [10] proposed parameters, such as source, appearance, viscosity or *M*r, particle size, degree of deacetylation (DD), solubility in aqueous 0.5% acetic acid, moisture content, and ash for the specification of chitosan used in medical, cosmetic, standard, and industrial applications. In addition to the three most commonly used parameters of *M*r, DD, and solubility, parameters, such as heavy metal, microbial, protein, and pyrogen contents, are considered to be important, and need to be specified for medical usage.

The physicochemical properties and bioactivity of chitosan, such as aggregation of whole blood, washed erythrocytes and platelets in platelet-rich plasma [11], vaccine adjuvant activity [12], antioxidant activity, antimicrobial activity, enzyme and DNA binding ability and protection, cholesterol, lipid and metal binding capacity, drug delivery property, ionic conductivity and thermal stability of biosensors, rheological property, chain flexibility, mechanical property and pore size of membranes and microcapsules, and water-holding capacity [2,3,13,14,15], depend on intrinsic factors such as the DD, *M*r, and polydispersity index (PDI).

The regeneration process is a frequently employed and practical measure to remove unwanted contaminants [16,17,18,19,20,21], to improve the solubility [9,22,23,24,25], or change to physicochemical properties [9,21,24]. These functions have been revealed to be dependent, not only upon the chemical structure, but also on the regeneration method.

The principle of the regeneration process is dissolving the chitosan in an acid solution in order to remove unwanted materials. Acids used include acetic acid [10,16,17,18,19,20,21], formic, propionic, lactic, citric, and sulfuric acid [21]. Next is precipitation by alkali neutralization, such as by using 5% aqueous sodium hydroxide solution at room temperature [19,23,26] or alkali/alcohol/water mixtures [18], or by dialysis [9].

The regeneration procedure is not only employed to remove the unwanted contaminants but also is demonstrated to increase bioactivity, such as immune adjuvant activity, and among different DD regenerated chitosans obtained, the best performance was shown at around 70% of deacetylation [19]. The regenerated chitosan can elevate the absorption capacity of anionic dye [27], or fat or the orange II dye [28]. The absorption of fat and the orange II dye was 1.5–2.0 times higher with regenerated chitosan, with 87% and 96% DD, than that of the 75% DD counterpart, but water absorption capacity was just the opposite. Dutkiewicz *et al.* [26] reported that regenerated krill chitosan with weight-average molecular weights (*M*w) of 800 kDa shows whole blood clotting time two times longer than that of silica gel. The above-mentioned literature indicated that those enhanced functional properties may be attributed to difference in solubility, *M*r, and DD, which in turn resulted in different content of positively charged amino groups in an acid solution between original and regenerated chitosan. Protonated amino groups may result in better immune adjuvant activity and fat and anionic dye absorption. However, up to now, the regenerated conditions used and the effect of characters of chitosan, such as the *M*r, DD on the yields, and physicochemical of regenerated products have not been systematically explored.

The objective of this study was to explore the effects of the characters of chitosan used and regeneration conditions on the yield and physicochemical characteristics of regenerated products. The different DDs and *M*w of chitosans were dissolved in 0.1 and 1.0 M acetic acid, respectively, then chitosan was precipitated with 8 N NaOH, followed by washing, neutral and freeze drying to get the regenerated products. The physicochemical properties, such as *M*w, PDI, DD and crystallinity index of regenerated products, were measured.

## 2. Results and Discussion

### 2.1. The Yield

Results in Table 1 show that the yield of R-2 was higher than R-1 and the yield of R-4 was higher than R-3. This may be attributed to using a higher concentration of acetic acid (1.0 M) will dissolve more chitosan than using a lower concentration (0.1 M) [29], and the insoluble materials were filtered and discarded prior to the solution proceeding to the regeneration procedure; therefore, the yield of R-2 was higher than R-1, thus R-4 was higher than R-3. The yield of R-4 was higher than R-2, whereas the yield of R-3 was higher than R-1. It indicated that the higher the DDs of chitosan used, the higher the yield of regenerated chitosan was obtained. The result was similar to that of Chen and Liu [9]. This may be due to the fact that the solubility of lower-DD or higher-*M*r chitosan was lower than that of the higher-DD or lower-*M*r one, respectively [14,23]. The reasons for using higher DD chitosan that ends up with a higher yield than when using lower DD chitosan might be because the insoluble materials were filtered and discarded prior to the solution that proceeded to the regeneration procedure.

### 2.2. The Ash Content and Bulk Density

Changes of ash content and bulk density of regenerated chitosans are listed in Table 1. Ash content decreased significantly after regeneration. It decreased from 1.94% of O-1 to 0.35% and 0.33% for R-1 and R-2, respectively, and from 1.75% of O-2 to 0.29% and 0.27% for R-3 and R-4, respectively. Results indicated that the different acid concentration treatments and/or different DDs did not differ significantly in ash. Trung [21] reported there was an insignificant difference in ash content removal among different acid treatments. It is conceivable that some minerals were not removed during the extraction of the original chitosan from raw material because they were enclosed in the interior of the solid material and protected against hydrochloric acid used for deminerization. In the regeneration procedure, the chitosan is completely dissolved, the minerals will be freed and will react with acid solvent. Thus, ash measurement is an indicator of the effectiveness of the regeneration step for removal of impurities.

**Table 1:** Effects of the characters of chitosans used and regeneration conditions on the yield and physicochemical characteristics of regenerated products.

Sample			Characteristics				
DD (%)	Acetic Acid (M)	Product *	Yield (%)	Ash (%)	Bulk Density (g/cm^3^)	*M*w (kDa)	PDI
61.7	-	O-1	-	1.94 ± 0.15 ^a^	0.55 ± 0.01 ^a^	176 ± 3.3 ^a^	2.07 ± 0.03 ^a^
	0.1	R-1	53.54 ± 0.31 ^a^	0.35 ± 0.03 ^b^	0.49 ± 0.01 ^b^	155 ± 3.5 ^b^	1.72 ± 0.02 ^b^
	1.0	R-2	54.73 ± 0.42 ^b^	0.33 ± 0.03 ^b^	0.48 ± 0.01 ^b^	150 ± 1.4 ^c^	1.73 ± 0.03 ^b^
94.9	-	O-2	-	1.75 ± 0.13 ^x^	0.55 ± 0.01 ^x^	97 ± 1.7 ^x^	2.09 ± 0.03 ^x^
	0.1	R-3	54.63 ± 0.33 ^x^	0.29 ± 0.04 ^y^	0.49 ± 0.01 ^y^	89 ± 1.0 ^y^	1.73 ± 0.02 ^y^
	1.0	R-4	58.69 ± 0.25 ^y^	0.27 ± 0.03 ^y^	0.49 ± 0.01 ^y^	88 ± 1.1 ^y^	1.78 ± 0.03 ^y^

^a–c^ Reflect mean values (*n* = 3) and followed by the difference superscripts within the 61.7% DD chitosan are significantly different (*p* < 0.05); ^x–y^ Reflect mean values (*n* = 3) and followed by the difference superscripts within the 94.9% DD chitosan are significantly different (*p* < 0.05); * O-1 and O-2 represent original 61.7% and 94.9% DD chitosan, respectively; R-1 and R-2 represent O-1 dissolving in 0.1 and 1.0 M acetic acid to obtain regenerated chitosans, restectively; R-3 and R-4 represent O-2 dissolving in 0.1 and 1.0 M acetic acid to obtain regenerated chitosans, respectively.

The change in bulk density content decreased significantly after regeneration. It decreased from 0.55 g/cm^3^ of O-1 to 0.49 and 0.48 g/cm^3^ for R-1 and R-2 respectively, and from 0.55 g/cm^3^ of O-2 to 0.49 and 0.49 g/cm^3^ for R-3 and R-4 respectively. Results indicated that the different acid concentration treatments and different DDs differed insignificantly in bulk density. The results were similar to Trung [21]. The reduction in ash contained might result in lower bulk density and elevate the purity of the resulted products; therefore, the purities of regeneration chitosans were better than the original ones. This may be one of the reasons why regenerated chitosan shows better performance in medical, cosmetics and biochemical studies than the original chitosan [16,17,19,22,23,26,27,28].

### 2.3. The Mw and PDI

**Figure 1:**
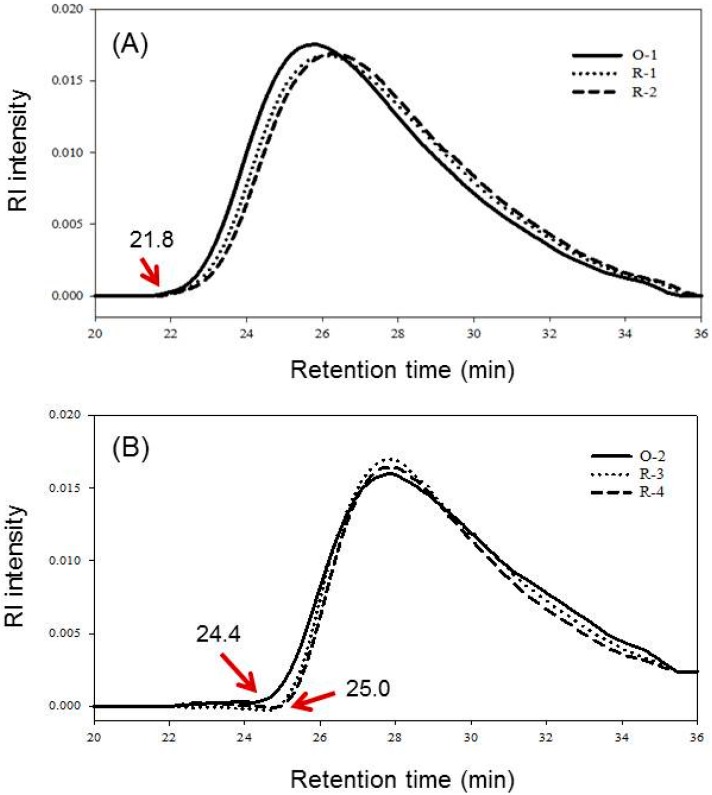
The SE-HPLC elution patterns of chitosans and regenerated chitosans. (**A**) 61.7% DD chitosan; (**B**) 94.9% DD chitosan. Samples O-1, R-1, R-2, O-2, R-3, and R-4, respectively, are described in Table 1.

Figure 1 shows the elution patterns of SE-HPLC of original chitosans (O-1 and O-2) and their regenerated chitosans (R-1, R-2, R-3 and R-4). The *M*w and PDI of these samples were calculated and listed in Table 1. *M*w decreased after regeneration of both high and low DD chitosans. It decreased from 176 kDa of O-1 to 155 and 150 kDa for R-1 and R-2, respectively, and from 97 kDa of O-2 to 89 and 88 kDa for R-3 and R-4, respectively. After regeneration, *M*w of regenerated chitosan decreased, possibly due to material loss during the filtering insoluble materials, precipitation process and sieving, as well as acid hydrolysis. Decreases in *M*w after being regenerated was more significant of Low DD chitosan (R-1: 11.9%, R-2: 14.8%) than high DD ones (R-3: 8.2%, R-4: 9.3%). The reasons were similar to the previous one just mentioned, *i.e.*, due to different solubilities of O-1 and O-2 chitosans in acetic acid solution, precipitation, and collection process. However, from the size exclusion high performance liquid chromatography (SE-HPLC) elution curve, the decrease in *M*w of high DD chitosan might be more significant than the low DD one, *i.e.*, decrease in higher *M*w portion was faster in the beginning of elution curve (Figure 1). This is illustrated from the difference of elution curve at starting increase points (arrow) between R-3 and R-4 (both at 25.0 min) to O-2 (at 24.4 min) were more significant than that of the peak in R-1 and R-2 to O-1 (all at 21.8 min). It has been assumed that a high DD chitosan molecule has an extended contour due to more electrostatic force between –NH_3_^+^ groups, and the glycosidic linkage is easier to access by H^+^ hydrolysis reaction [30].

Results in Figure 1 show the molecular weights distribution of low and high DD chitosans (O-1 and O-2, respectively) and their regenerated chitosans (R-1, R-2, R-3, and R-4). The PDIs of both original chitosans were similar to each other, and their regenerated ones were also similar to each other. The distribution of 61.7% DD chitosan and its regenerated products showed that the high-*M*r side (the left-hand side of peak) is steeper, whereas the low-*M*r side (the right-hand side of peak) is tailing. After regeneration, the elution curves of R-1 and R-2 were shift to right-hand side of O-1 (Figure 1A). This indicated that the high-*M*r fractions (21.8–26.0 min) of R-1 and R-2 decreased, so the retention time were longer than that of O-1, consequently, low-*M*r fractions (26.0–35.5 min) increased. The distribution of 94.9% DD chitosan and its regenerated products showed that the high-*M*r side is steeper (24.0–28.0 min), whereas the low-*M*r side (28.0–35.5 min) is tailing (Figure 1B), similar to Figure 1A. However, the proportion of medium-*M*r fractions (27.0–29.0 min) increased significantly after regeneration (Figure 1B). Furthermore, the PDI decreased both after regeneration for high or low DD chitosans used. It decreased from 2.07 of O-1 to 1.72 and 1.73 for R-1 and R-2, respectively, and from 2.09 of O-2 to 1.73 and 1.78 for R-3 and R-4, respectively. Results indicated that the PDI of regenerated products narrows down after the regeneration process.

The merits of the regeneration process can narrow down the PDI have not been reported. The result is unprecedented. The reasons that PDI of chitosan narrows down after regeneration may be due to different solubilities of 61.7% and 94.9% DD chitosans in 0.1 or 1.0 M acetic acid, and different susceptibilities to acid hydrolysis of low and high DD chitosans [31]; it also may be due to the precipitation process and sieving, washing, and collection to get the regenerated products. Therefore, the elution patterns of regenerated products changes; the ratio of medium-*M*r fractions increased and PDI decreased. The regeneration procedure of precipitation process and sieving, washing, and collection and it affects the *M*w and PDI has not been studied. However, those steps should affect the *M*w and PDI of the regenerated product. Therefore, they should be explored in the future studies.

### 2.4. FTIR Spectrum

**Figure 2:**
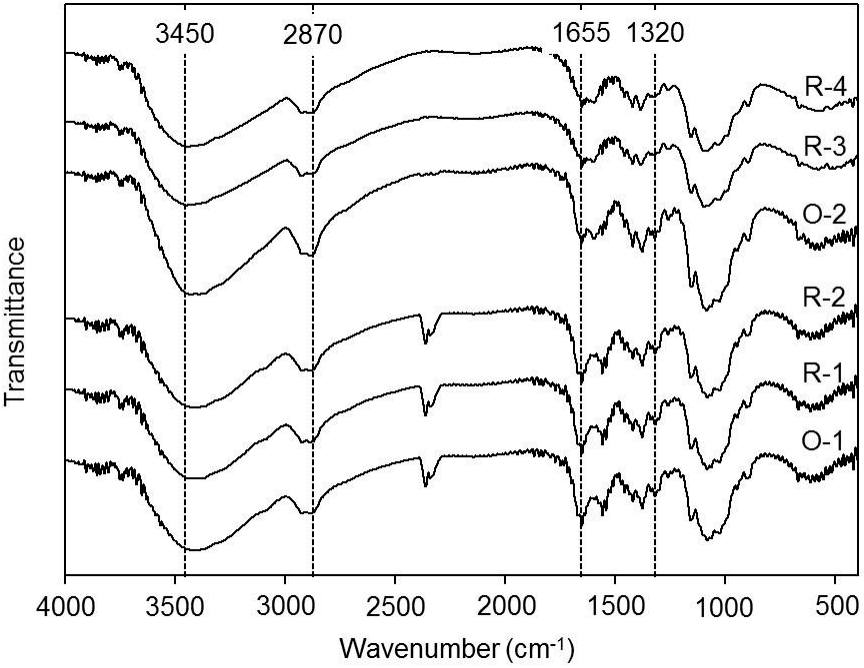
Fourier transform infrared spectra (FTIR) of chitosans and regenerated chitosans. Samples O-1, R-1, R-2, O-2, R-3, and R-4, respectively, are described in Table 1.

In the literature, the OH stretching band at 3450 cm^−1^ [32]; the CH stretching bands within 2870–2880 cm^−1^ [33]; the amide I band at 1650–1655 cm^−1^; the amide II band at 1550–1555 cm^−1^; the amide III band at 1315–1320 cm^−1^ [34] have been reported. Results in Figure 2 show that the FTIR spectra of the above-mentioned functional absorption bands have not changed significantly after regeneration. The functional groups on the backbone of different regenerated chitosans either from lower DD chitosans (O-1 to R-1 and R-2) or higher DD chitosans (O-2 to R-3 and R-4) were the same as the original chitosans. Results imply that the regenerated process used in this report did not result in changes of functional groups and DD of polymer chains, suggesting that the acetamido groups were stable during the regenerated treatment. Therefore, the regeneration method is a very good practice to produce final products with higher purity at the same time the functional groups and DD of the chitosan can be preserved.

### 2.5. The Crystallinity

**Figure 3:**
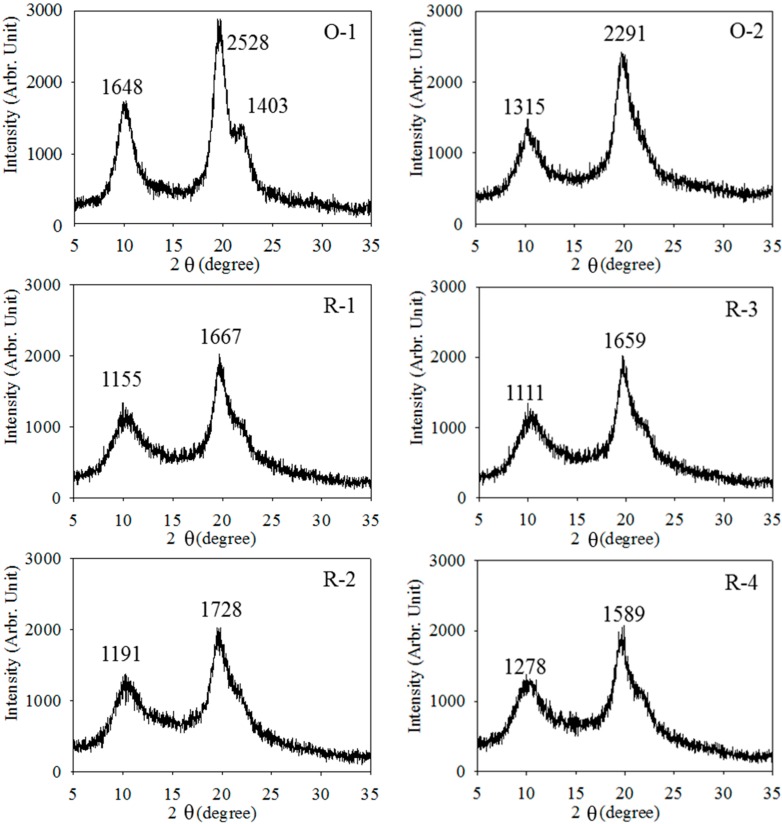
X-ray diffraction spectra of chitosans and regenerated chitosans. Samples O-1, R-1, R-2, O-2, R-3, and R-4, respectively, are described in Table 1.

The degree of crystallinity for chitosans (O-1 and O-2) and regenerated chitosans (R-1, R-2, R3, and R4) were evaluated through X-ray powder diffraction method. Webster *et al*. [35] reported that the diffraction pattern of chitin which exhibits four diffraction peaks at 9°, 12°, 19°, and 26°. The peaks at 10° and 20° are the two prominent peaks in the diffraction pattern, which confirms the partial crystallinity of the polymer. Ogawa [36] reported four crystalline polymorphs, they are tendon, annealed, L-2 and I-2. The tendon and L-2 crystals are hydrated, *i.e.*, water molecules are incorporated. However, the annealed polymorph is anhydrous. The hydrated polymorph (tendon or L-2) showed a strong equatorial reflection spot at 2θ of around 10°, whereas, the anhydrous crystal (annealed) exhibited a strong spot at around 15°, L-2 showed a strong reflection at 2θ at 10.6° and no diffraction spot at around 15°. But I-2 showed diffraction spot at both 2θ of 10.7° and 15.4°. Chitosan after deacetylated to 61.7% (O-1), only three broad peaks appeared at 10°, 20°, and 22°, this may be due to the deacetylaction reaction occurred under high temperature and the strong alkaline condition, displacing the acetamido group to amine group [35]. The resulted showed a similar diffraction pattern that of the hydrated L-2 polymorph of chitosan. After deacetylated to 94.9% (O-2) and regenerated to R-1, R-2, R-3, and R-4, resulted showed that they are hydrated L-2 polymorph, however, only two broad peaks appeared at 10° and 20°, as shown in Figure 3. The results were similar to those of Ogawa [36]. However, Trung [21] reported the diffraction of most regeneration chitosan showed only one major peak at approximately 2θ = 20°. The crystallinity intensity decreased significantly after regeneration. Results in Figure 3 show that I_020_ (at 2θ = 10°) decreased from 1648 of O-1 to 1155 and 1191 for R-1 and R-2, respectively, and from 1315 of O-2 to 1111 and 1278 for R-3 and R-4, respectively, whereas I_110_ (at 2θ = 20°) of X-ray intensity decreased from 2528 of O-1 to 1667 and 1728 for R-1 and R-2, respectively, and from 2291 of O-2 to 1659 and 1589 for R-3 and R-4, respectively of X-ray intensity. Results indicated that the structure of regenerated chitosans (R-1, R-2, R-3, and R-4) and their counterparts (O-1 and O-2) differed significantly in crystalline intensity. This is may be due to the fact that original chitosan at 2θ = 10° or 20° decreased with the increase of DD [37] or structure change. However, the DD between original and regenerated chitosans did not change significantly (Figure 2 and Table 1). Thus, the decreases of I_020_ and I_110_ of regenerated chitosans should be structure change. On the other hand, the X-ray spectra among regenerated chitosans were similar, which suggest that the same regeneration process including precipitation, washing, neutral and freeze drying might cause the similar structure of regenerated chitosans.

Results in Table 2 shows that CrI_110_ decreased from 80.99% of O-1 to 66.39% and 62.33% for R-1 and R-2, respectively, and decreased from 72.53% of O-2 to 65.71% and 59.11% for R3 and R4, respectively. Results indicated that the regenerated chitosans had a lower crystallinity index than the original chitosans.

**Table 2:** Crystalline characteristics of various chitosan and regenerated chitosan products.

Product	2θ (degree)			C_r_I_110_ (%)
O-1	10.00	19.94	22.02	81.0 ± 0.6 ^a^
R-1	10.06	19.96	-	66.4 ± 0.7 ^b^
R-2	10.02	19.94	-	62.3 ± 1.2 ^c^
O-2	10.08	20.24	-	72.5 ± 0.6 ^x^
R-3	10.12	20.20	-	65.7 ± 1.1 ^y^
R-4	10.10	20.20	-	59.1 ± 0.2 ^z^

^a–c^ Reflect mean values (*n* = 3) and followed by the difference superscripts within the 61.7% DD chitosan are significantly different (*p* < 0.05); ^x–z^ Reflect mean values (*n* = 3) and followed by the difference superscripts within the 94.9% DD chitosan are significantly different (*p* < 0.05). Samples O-1, R-1, R-2, O-2, R-3, R-4, respectively, are described in Table 1.

At I_110_, the crystallinity intensity of R-1 and R-2 those regenerated from O-1 also R-3 and R-4 that regenerated from O-2 were close. However, the crystallinity intensity of O-1 and O-2 are different from each other; furthermore, they are different from the regenerated product derived from them. The difference in I_110_ between O-1 and O-2 should be due to the deacetylation process. The similarity in I_110_ among R-1, R-2, R-3, and R-4 may be due to similar processes of dissolving by acetic acid and alkali precipitation, then washing, neutral, freeze drying, which therefore, end up with a similar molecular arrangement and, thus, similar crystallinity intensity.

## 3. Experimental Section

### 3.1. Materials

α-Chitin was purchased from the OHKA enterprises Co., Ltd. (Kaohsiung, Taiwan). Acetic acid, sodium acetate, sodium azide, sodium chloride, and potassium bromide were purchased from the Sigma-Aldrich Co. (St. Louis, MO, USA). Hydrochloric acid and sodium hydroxide were purchased from Merck and Co., Inc. (Darmstadt, Germany). Pullulan standards (for SE-HPLC calibration) were purchased from Showa Denko (Tokyo, Japan).

### 3.2. Preparation of Chitosan

Chitin powder was passed though sieves of 40–60 mesh, and then was alkali-treated (50% NaOH) at 140 °C for 1 or 3 h to get different DD chitosan. Chitosan was washed until neutral and dried at 50 °C to get the final product [5].

### 3.3. Preparation of Regenerated Chitosan

The modified procedure of Chen and Liu [9] was used to prepare the regenerated chitosans. Chitosans with different DD were dissolved in 0.1 or 1.0 M acetic acid to make 1% solutions, stirred for 10 h then filtered through filter paper (Toyo No. 1, 90 mm, Toyo Roshi Kaisha, Tokyo, Japan) to remove insoluble materials. One liter of 1% chitosan-acetic acid solution was added to two liters of 8 N NaOH solution to precipitate the chitosan. The precipitates were collected with a 325-mesh sieve and washed with distilled water until neutral. The precipitates were freeze dried to obtain the product that was ground and sieved through a 40–60 mesh size to get regenerated chitosans.

### 3.4. Determination of DD

Infrared spectrometry was used to determine the DD of the chitosan or regenerated chitosan [38]. Chitosan or regenerated chitosan powder was sieved through a 200 mesh and then mixed with KBr (1:100), dried at 60 °C for 3 days to prevent interference of the effect of water molecules on the peak of hydroxyl band in FTIR measurements, and pressed into a pellet. The absorbance of amide I (1655 cm^−1^) and the hydroxyl band (3450 cm^−^^1^) were measured using a Bio-Rad FTS-155 infrared spectrophotometer (Hercules, CA, USA). The band of the hydroxyl group at 3450 cm^−1^ was used as an internal standard to correct for disc thickness and for differences in chitosan concentration when making the KBr disc. Triplicate measurements were averaged and used to calculate the DD using the following equation:

(1) DD = 100 − (A_1655_/A_3450_) × 115

here, A_1655_ and A_3450_ were the absorbance at 1655 and 3450 cm^−1^, respectively.

### 3.5. Determination of Molecular Weight and PDI

The weight-average molecular weight (*M*w), number-average molecular weight (*M*n) and polydispersity index (PDI = *M*w/*M*n) of samples were measured by size exclusion high performance liquid chromatography (SE-HPLC) [14]. A column (7.8 mm × 30 cm) packed with TSK gel G4000 PW_XL_ and G5000 PW_XL_ (Tosoh Co., Ltd., Tokyo, Japan) was used. The mobile phase consisted of 0.2 M acetic acid/0.1 M sodium acetate and 0.008 M of sodium azide. A sample concentration of 0.1% (*w*/*v*) was loaded and eluted with a flow rate of 0.6 mL/min by an LDC Analytical ConstaMetric 3500 pump (Thermo Scientific, Waltham, MA, USA). The elute peak was detected by an RI detector (Gilson model M132, Gilson, Middleton, WI, USA). The data were analyzed by Chem-Lab software (SISC 3.0, Scientific Information Service, Taipei, Taiwan). Pullulan standards with different *M*r values were used as markers. The *M*r values of the samples were calculated from the pullulan calibration curve with Chem-Lab software.

### 3.6. Determination of Bulk Density

Chitosan or regenerated chitosan powder was assayed for its bulk density as described by Cho *et al*. [39]. One gram of chitosan or regenerated chitosan (40–60 mesh particle size) was placed in a 15-mL tapered graduated centrifuge tube, vibrated on a vortex mixer for 1 min, and packed by gently tapping the tube on the bench top 10 times. The volume of the sample was recorded. The procedure was repeated three times for each sample, and the bulk density was computed as grams per milliliter of the sample.

### 3.7. Determination of Crystallinity

The crystallinity of chitosan or regenerated chitosan was measured by a Miniflex Rigaku X-ray diffractrometer, using Ni filtered Cu Ka radiation generated at 30 kV and 10 mA at a scanning speed of 2° 2θ/min within a range from 5° to 30°. The crystallinity index was also used, based on the method proposed by Zhang *et al*. [37]. It consisted of measuring the maximum peak intensity, I_110_, at 2θ = 20° of the (110) lattice diffraction and that of the amorphous diffraction, I_am_, at 2θ = 16°. The crystallinity index (CrI_110_) was calculated using the following formula:

(2) CrI_110_ = [(I_110_ − I_am_)/I_110_] × 100

### 3.8. Determination of Ash

The ash content was deduced from the difference in weight before and after a thermal treatment of the product in an electric furnace. The crucible containing the dry sample was placed in an electric furnace at 600 °C for 6 h. Ash content was estimated by ignition of a chitosan or regenerated chitosan sample in an electric furnace and quantization of the ash by gravimetric analysis [40].

### 3.9. Statistical Analysis

All experiments were carried out in triplicate and average values or means (standard deviations) reported. Mean separation and significance for correlation were analyzed using the SPSS (Statistical Package for Social Sciences, SPSS Inc., Chicago, IL, USA) software package.

## 4. Conclusions

Higher DD of original chitosan and/or higher concentration of acetic acid used resulted in higher purity, however, the bulk density, *M*w, PDI, and crystallinity intensity of regenerated chitosan were not affected.

The regeneration process could increase the purity, manipulate the *M*w and narrow down the PDI of regenerated chitosan and lower the crystallinity intensity, and bulk density, whereas the DD, structure and functional groups on chitosan molecules were preserved. These changes would be beneficial to chitosan for use in the biomedical field. Therefore, the regeneration process has a bright future in the developing areas of medical applications.
